# Nanostructured In_3_SbTe_2_ antennas enable switching from sharp dielectric to broad plasmonic resonances

**DOI:** 10.1515/nanoph-2022-0041

**Published:** 2022-04-25

**Authors:** Andreas Heßler, Sophia Wahl, Philip Trøst Kristensen, Matthias Wuttig, Kurt Busch, Thomas Taubner

**Affiliations:** Institute of Physics (IA), RWTH Aachen University, 52074 Aachen, Germany; Humboldt-Universität zu Berlin, Institut für Physik, 12489 Berlin, Germany; Max-Born-Institut, Max-Born-Straße 2A, 12489 Berlin, Germany

**Keywords:** dielectric metasurfaces, infrared active metasurfaces, phase-change materials, plasmonic metasurfaces, resonance switching

## Abstract

Phase-change materials (PCMs) allow for non-volatile resonance tuning of nanophotonic components. Upon switching, they offer a large dielectric contrast between their amorphous and crystalline phases. The recently introduced “plasmonic PCM” In_3_SbTe_2_ (IST) additionally features in its crystalline phase a sign change of its permittivity over a broad infrared spectral range. While optical resonance switching in unpatterned IST thin films has been investigated before, nanostructured IST antennas have not been studied, yet. Here, we present numerical and experimental investigations of nanostructured IST rod and disk antennas. By crystallizing the IST with microsecond laser pulses, we switched individual antennas from narrow dielectric to broad plasmonic resonances. For the rod antennas, we demonstrated a resonance shift of up to 1.2 µm (twice the resonance width), allowing on/off switching of plasmonic resonances with a contrast ratio of 2.7. With the disk antennas, we realized an increase of the resonance width by more than 800% from 0.24 µm to 1.98 µm while keeping the resonance wavelength constant. Further, we demonstrated intermediate switching states by tuning the crystallization depth within the resonators. Our work empowers future design concepts for nanophotonic applications like active spectral filters, tunable absorbers, and switchable flat optics.

## 1 Introduction

Infrared light plays an important role in everyday life since it is tightly interwoven with modern technology, from telecommunications over thermal imaging to medical diagnostics. Infrared optical instruments are normally bulky and only have one given function at a time. Driven by the rapid development of technology, there is an increasing need for lightweight and compact infrared optical devices with adaptable integrated functionalities, e.g., for mobile phone technology or autonomous driving.

Developing such ultracompact optical devices is subject of current research in the field of infrared nanophotonics, where infrared light is manipulated at subwavelength scales. Metasurfaces have emerged as a promising solution [1], [2], [3], [4]. They are only as thick as a fraction of the wavelength and consist of periodically arranged subwavelength nanoantennnas whose individual and collective resonances together govern the metasurface’s optical response. The nanoantennas can either be plasmonic [2, 4] or dielectric [3]. In plasmonic metasurfaces, antennas consist of metals like gold. The free electrons of the metals can take up part of the electromagnetic energy, allowing very high field confinement to deeply subwavelength regions at the outside of the antennas at the cost of increased optical losses [5], which leads to relatively broad antenna resonances. This makes plasmonic metasurfaces especially suitable for applications such as broadband optics [6], energy harvesting [7] or (bio)chemical sensing [8]. In dielectric metasurfaces, the antennas consist of lossless dielectrics such as silicon. Thus, there are only very small optical losses, leading to relatively narrow antenna resonances. The electromagnetic fields are confined within the dielectric antennas and thus to larger volumes than for plasmonic antennas. Therefore, dielectric metasurfaces are well-suited for applications in planar optics like metalenses [9] or meta-holograms [10].

The concept of metasurfaces, while originally only enabling static functionality fixed after fabrication [11], has now evolved towards active optical devices [12, 13] by employing various tuning mechanisms like stretchable substrates [14, 15], electrostatic biasing of semiconductors [16], graphene [17] or liquid crystals [18], or using the phase-transition material VO_2_ [19]. Notably, metallic polymer antennas were recently demonstrated in the infrared spectral range by Karst et al. [20], where electric biasing allowed volatile on/off switching of a plasmonic resonance to realize reconfigurable beam steering. All of these tuning mechanisms are volatile, i.e. the change of optical properties only persists as long as an external stimulus is applied. The non-volatile switching of infrared nanoantennas between dielectric and plasmonic optical properties has not yet been explored.

For non-volatile tuning, phase-change materials (PCMs) have emerged as promising active metasurface components [21], [22], [23], enabling tuning of absorption [24], light focusing [25, 26], beam steering [25, 27] and optical filtering [28]. Their crystalline state is characterized by an octahedral-like atomic arrangement where adjacent atoms form *σ*-bonds of p-orbitals. These bonds are only occupied by half an electron pair, resulting in an increased electron delocalization and polarizability. Deviations from the perfect octahedral configuration lead to a decrease in electron delocalization and polarizability [29, 30]. This is the case in the amorphous phase where the material properties resemble those of ordinary covalently bonded solids. The special type of bonding in the crystalline phase is distinctly different from other bonding mechanisms like covalent or ionic bonding and is termed “metavalent bonding” [31], [32], [33]. Thus, the change in chemical bonding from covalent in the amorphous phase to metavalent in the crystalline phase gives rise to a pronounced optical contrast between the two phases.

A PCM can be switched between its two phases via thermal annealing [24, 34, 35], electrical [36, 37] or optical pulses [38, 39]. For crystallization, the PCM is heated above its crystallization temperature (approx. equal to the glass transition temperature) and for amorphization, it is heated above its melting temperature and then quenched rapidly (with rates >10^9^ K/s). The optical switching with a pulsed laser enables direct writing of dielectric antennas [40] and holographic metasurfaces [41] into a thin PCM layer and optical programming of PCM metasurfaces [42, 43] where each nanoantenna can be switched individually to different states.

In the infrared spectral range, the real part of the permittivity, *ε*′, of PCMs is generally positive in both phases, resulting in dielectric optical properties. There is, however, a small spectral window for the PCM Ge_2_Sb_2_Te_5_ (GST) in the visible spectral range where *ε*′ < 0 in the crystalline phase due to electronic interband transitions [44], although the optical losses are relatively large in the amorphous phase, too.

**Figure 1:**
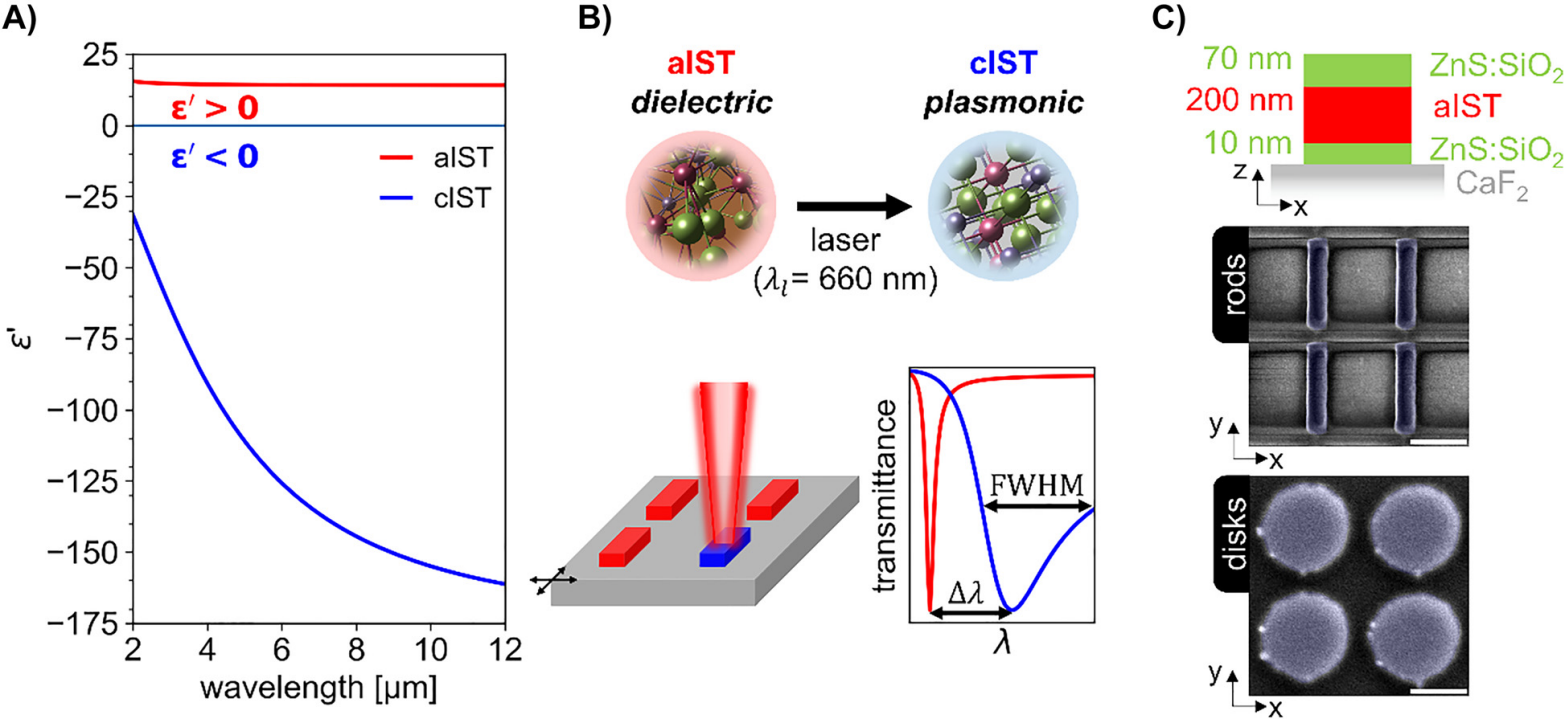
Optically switching nanostructured In_3_SbTe_2_ (IST) antennas between dielectric and plasmonic response. (A) The real part of the permittivity, *ε*′, of IST changes its sign from positive in the amorphous phase (aIST, red) to negative in the crystalline phase (cIST, blue). (B) A pulsed laser can crystallize the IST and change its infrared optical properties from dielectric (*ε*′ > 0) to plasmonic (*ε*′ < 0). Nanostructured IST-antennas can then be switched between dielectric (aIST, red) and metallic (cIST, blue). This changes the type of the infrared resonances from sharp Mie resonances (aIST) to broad plasmonic resonances (cIST), significantly tuning both the shift Δλ and the width FWHM of the resonance. (C) Sketch of the layer stack of the investigated samples. SEM images of nanostructured IST rod and disk antennas fabricated with focused ion beam milling. The rods and disks are marked with blue-colored transparent overlays as guide-to-the-eye. The scale bars equal 1 µm. Depending on the geometry of the antennas and arrays, either both Δλ and FWHM (rods) or just FWHM (disks) can be tuned.

For application in the infrared spectral range, a different material is needed: Recently, the plasmonic PCM In_3_SbTe_2_ (IST) was introduced as a programmable nanophotonics material platform for the infrared [45]. In crystalline IST (cIST), there are not enough electrons available to share one electron between adjacent atoms, anymore. This corresponds to a metallic state, since the Fermi level now lies in the valence band. Because the electrons close to the Fermi level are less delocalized than in a good metal, materials like cIST are better denoted as ‘bad metals’. Thus, the conductivity of cIST (10^4^ S/cm [46]) is about 10–1000 times larger compared to that of crystalline GST [47] but still about 10 times smaller than that of conventional metals like gold and aluminum [45]. The real part of the permittivity, *ε*′, of IST is depicted in Figure 1A (see Figure S1, Supplementary Information, for the imaginary parts and a wider spectral range). In the amorphous phase (aIST), *ε*′ ≈ 14, whereas in the crystalline phase, it follows a Drude-like behaviour with *ε*′ < 0 throughout the whole infrared spectral range. Thus, IST’s optical properties can be switched between lossless dielectric in the amorphous phase and plasmonic (lossy) in the crystalline phase.

It has recently been demonstrated that local switching of IST thin films with laser pulses can be used to directly write, erase or reconfigure infrared plasmonic nanoantenna geometries to tune their antenna resonances [45, 48]. Additionally, it was shown that IST can be used in thin-film absorbers for smart infrared modulators [49] or with pre-patterned plasmonic metasurfaces to switch individual resonances on/off and to “solder” together neighboring antenna parts [45]. Patterning of IST itself into resonant nanoantennas and their optical switching have not been studied, yet. While nanostructured PCM antennas have been investigated before [26, 43, 44, 50], [51], [52], [53], this research was limited by the available PCMs to all-dielectric PCM designs or to the visible and near-infrared spectral ranges.

IST is ideally suited to combine the concepts of (lossy) plasmonic and lossless dielectric resonances on reconfigurable infrared metasurfaces, which consist of nanostructured IST antennas (see Figure 1B). By local optical switching with a pulsed laser, the resonances of individual nanoantennas can be non-volatilely switched between narrow Mie resonances (aIST) and broad plasmonic resonances (cIST), allowing tuning of the shift Δ*λ* and width FWHM of the resonance. Depending on the antenna and array geometry, Δ*λ* and FWHM can be tuned differently.

Here, we nanostructured a layer stack consisting of 10 nm ZnS:SiO_2_, 200 nm of IST and 70 nm capping made of ZnS:SiO_2_ on a CaF_2_ substrate into rod and disk antennas (see Figure 1C). We demonstrate a resonance shift of up to 1.2 µm (twice the resonance width) for the IST rods by crystallizing the IST with microsecond laser pulses. In the nanodisk geometry, we designed the antenna array geometry such that the broad plasmonic resonance gets “pinned” at the grating resonance, allowing a tuning of the FWHM by more than 800% while leaving the resonance wavelength almost constant. Our experimentally recorded spectra fit well to the performed numerical simulations. Additionally, we achieve intermediate switching states of the disk antennas by tuning the crystallization depth via optical switching.

## 2 Results

### 2.1 Tuning the resonance wavelength with IST rod antennas

**Figure 2:**
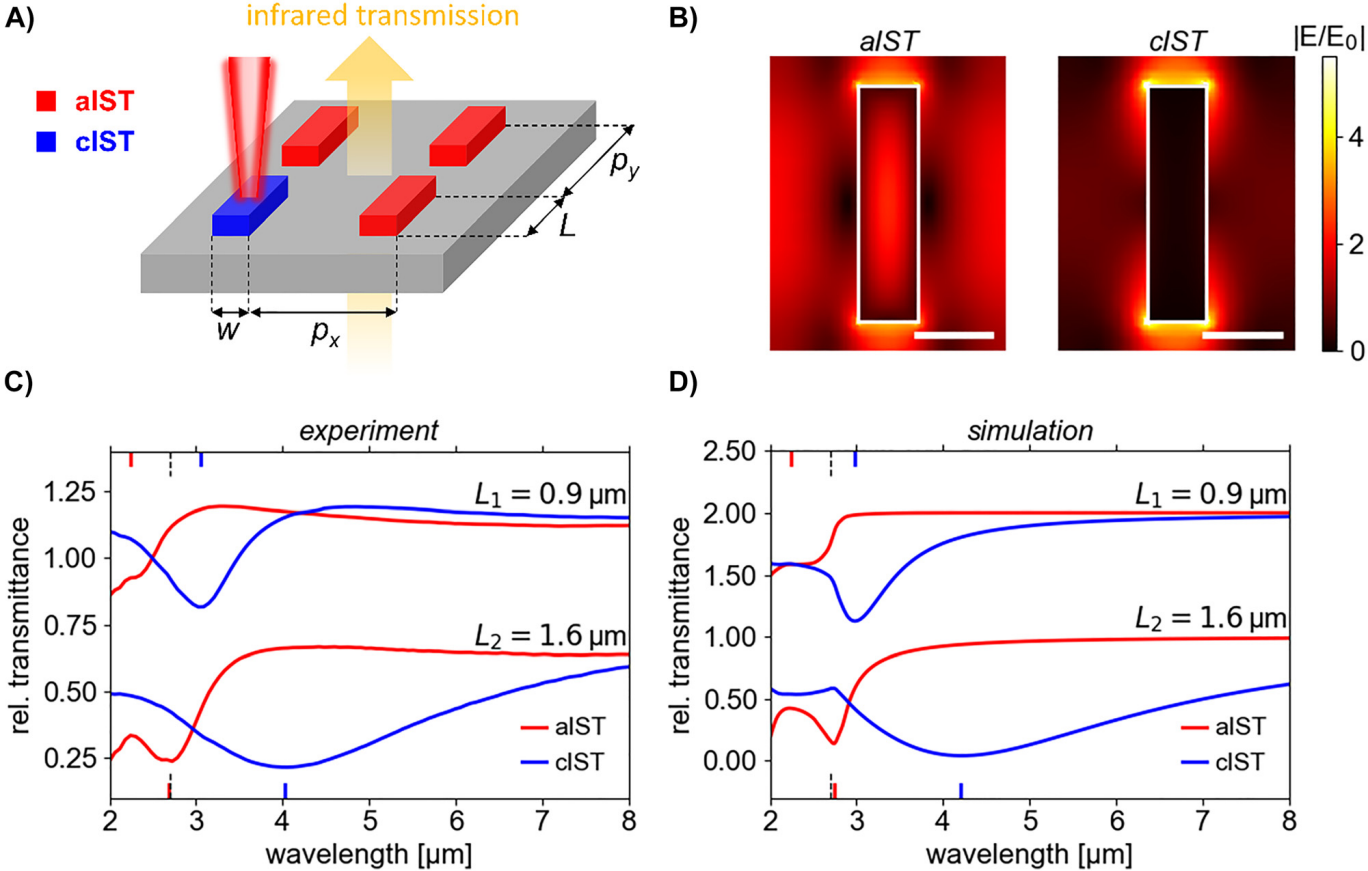
Resonance wavelength and width tuning with nanostructured IST rods antennas. (A) Sketch of the IST nanorod antenna array geometry, infrared illumination, and the optical crystallization of the antennas. (B) Simulated normalized absolute electric fields in the cross-section plane at half-height of the IST core of *L*_2_ = 1.6 µm long antennas for aIST (left) and cIST (right) in resonance. The scale bar is 500 nm long. The antenna boundary is shown with a white rectangle. (C) Measured and (D) simulated transmittance spectra of two rod antenna arrays with different lengths *L* in their aIST (red) and cIST (blue, optically crystallized) states. The dashed black line in the plots at the *x*-axis marks the position of the largest grating resonance through CaF_2_ (an average angle of incidence of 15° is assumed) and the colored ticks mark the transmittance minima. For clarity, the experimental spectra for *L*_1_ are shifted by 0.5 on the *y*-axis and the simulated spectra by 1.

In a first proof-of-principle experiment, we investigated IST rod antennas which were nanostructured by focused ion beam milling. Because the CaF_2_ substrate was electrically insulating, a 60 nm-thick layer of conductive polymer needed to be applied on top of the layer stack for the patterning to avoid accumulation of the positively charged ions (see **Materials and Methods**). The experiment is sketched in Figure 2A: The IST rods are arranged in a rectangular array of approx. 20 × 20 µm^2^ size, containing 12 × 10 antennas with periods $p_{x}=1.6$ µm and $p_{y}=2$ µm. The antennas themselves have a width of w=0.4 µm and a height equal to the total layer stack thickness (approx. 280 nm, see Figure 1C). We compared two antenna arrays with different antenna lengths of *L*_1_ = 0.9 µm and *L*_2_ = 1.6 µm. The IST rods are illuminated with infrared light polarized along their long antenna axis (*y*-direction) and transmission is measured in an FTIR microscope coupled to a spectrometer. By targeting individual rod antennas with microsecond laser pulses with a wavelength of 660 nm, the rod antennas can be switched from the amorphous (aIST) phase to the crystalline (cIST) phase (see **Materials and Methods** for more details on the experimental setup).

The simulated electric near fields in resonance of the IST rod antennas with length *L*_2_ = 1.6 µm are shown in Figure 2B. The images depict the cross-section (at a half-height of the IST core, *z* = 110 nm) of the absolute electric field normalized to the incident field (see **Materials and Methods** for details on the simulations). For aIST, a significant part of the field is inside of the dielectric antenna. In addition, the electric field is enhanced at the antenna tips because of the coupling to the neighboring rod antennas. While the absolute electric field is also largest at the antenna tips for cIST, it is practically completely expelled from the antenna body. Thus, the simulations confirm that the electric near-field distributions for the dielectric aIST and plasmonic cIST antennas are fundamentally different.

In Figure 2C, the measured transmittance spectra of the two different rod antenna arrays in their amorphous and crystalline phases are shown. The resonances can be recognized as minima in the measured relative transmittance. For aIST nanorods (red solid lines) with a length of *L*_1_ = 0.9 µm, the resonance is located at a wavelength of 2.25 µm. Because it overlaps with a higher order mode at smaller wavelengths, only the slope on the long-wavelength side of the transmittance minimum can be clearly discerned. For the longer aIST rods with a length of *L*_2_ = 1.6 µm, the resonance is located at a longer wavelength of 2.7 µm. For both antenna lengths, the resonances are highly influenced by the first order grating resonance [54] through the CaF_2_ substrate at about 2.7 µm (see black dashed line, an average angle of incidence of 15° is assumed): $\lambda_{\mathrm{Ca}F_{2}}=p_{x}\left(n_{\mathrm{Ca}F_{2}}+\sin\;\theta \right)\approx 1.7\;\mu m\cdot \left(1.4+\sin\;15{}^{\circ}\right)\approx 2.7$ µm. After optically crystallizing the rod antennas with the pulsed laser, the transmittance minimum is situated at significantly larger wavelengths (blue solid lines). For the smaller antennas with *L*_1_ = 0.9 µm, the resonance now occurs at a wavelength of 3.05 µm, resulting in a measured resonance shift of Δ*λ*_1_ = 0.8 µm. For the larger antennas with length *L*_2_ = 1.6 µm, the resonance for cIST is located at a wavelength of 4.09 µm, totaling a resonance shift of Δ*λ*_2_ = 1.34 µm. Compared to the width of the resonance of the aIST antennas, this results in tuning figure of merit of TFOM_2_ = Δ*λ*_2_/FWHM = 1.34 µm/0.6 µm = 2.23 for the longer antennas. This TFOM is significantly larger than previously realized with GeSbTe alloys (about 1 [55] to 1.8 [43]). The width of the resonance minimum in the amorphous phase cannot be determined. However, if the width of the resonance of the longer aIST is used as an approximation, we can estimate the TFOM of the shorter IST antennas to TFOM_1_ = 0.8 µm/0.6 µm = 1.33. The large TFOM values enable an on/off contrast ratio *c* = *T*(aIST)/*T*(cIST) (*T* is the respective rel. transmittance) in the plasmonic resonances of *c*_1_ = 2.2 for the antennas with length *L*_1_ and *c*_2_ = 2.7 for the antennas with length *L*_2_. This is significantly larger than the contrast ratio of approx. 1.4 achieved by Karst et al. [20] for the electrically switchable conductive polymer antennas.

Next, we compared our measurements to simulated transmittance spectra (see Figure 2D). They are averaged over the range of incident angles between 10° and 24° to approximate the light incidence through a Cassegrain objective. They agree well with the measured infrared spectra in Figure 2C. Since the antennas are simulated with in-plane periodic boundary conditions, forming an infinitely large antenna array, the grating resonances are generally slightly overemphasized compared to the measured spectra of the 12 × 10 antennas, even after the angle-averaging. The grating resonance at a wavelength of 2.7 µm (see black dashed line) can be seen best in the simulated spectra of the cIST rod arrays, where it appears as a sharp kink (especially for the longer antennas). In the simulated spectra of the aIST rod arrays, the grating resonance overlaps with the antenna resonance and cannot be easily discerned.

### 2.2 Tuning the resonance width with IST disk antennas

So far, we demonstrated that in nanostructured IST rod antennas the resonance wavelength and width can be significantly increased at the same time by optically crystallizing the IST. In the next step, we investigated if it is possible to just change the resonance width FWHM while keeping the resonance wavelength *λ*_res_ approximately constant, thus only tuning the resonance quality *Q* = *λ*_res_/FWHM.

For this, we employed an array of IST disk antennas. By correctly designing the antenna array period, the grating resonance wavelength can be used to “pin” the relatively broad plasmonic resonance to a certain wavelength. If the disk diameter is then chosen appropriately, the measured resonance position should be the same for the dielectric aIST antennas and the plasmonic cIST antennas (see Figure S2, Supplementary Information).

**Figure 3:**
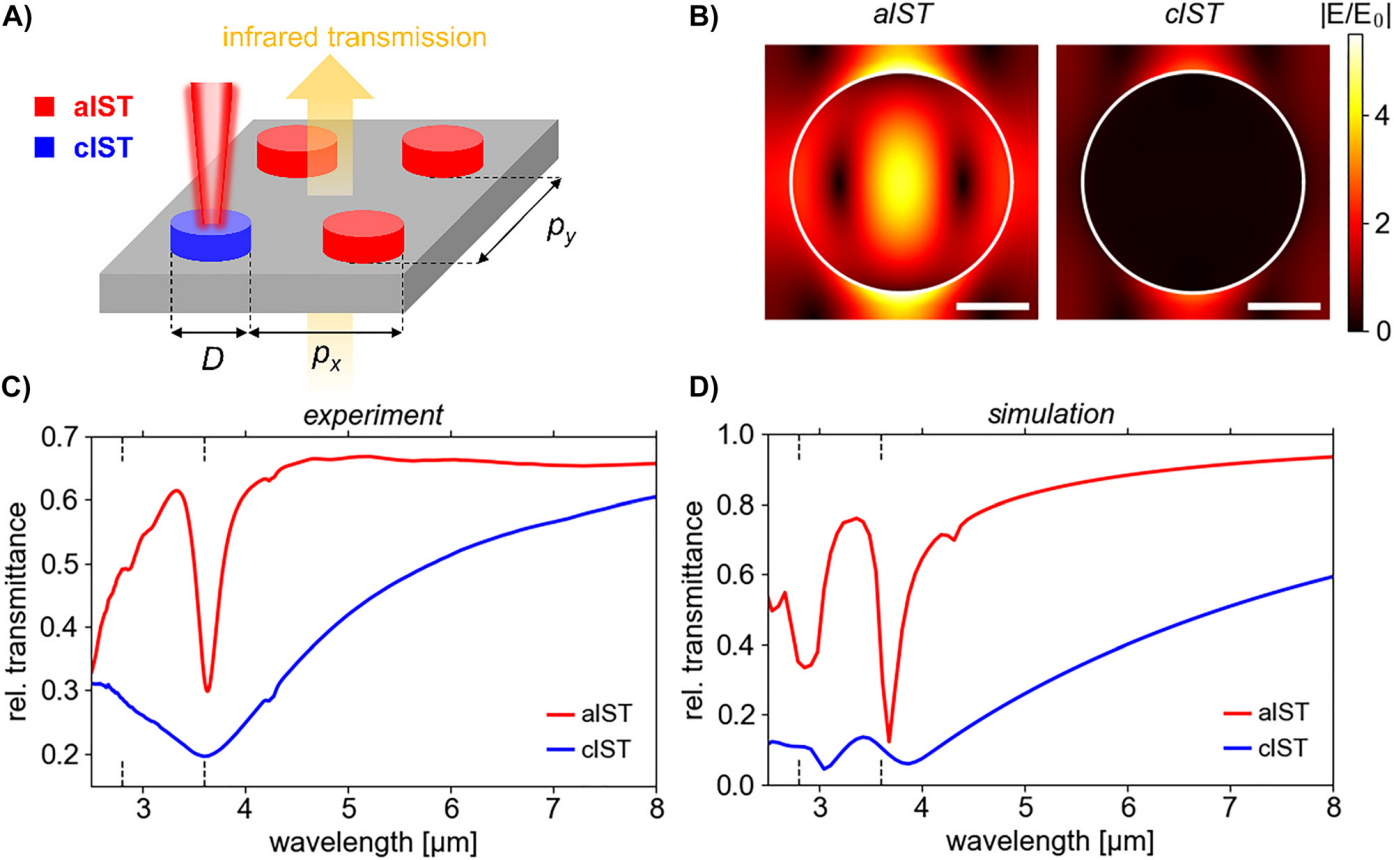
Resonance width tuning with nanostructured IST disk antennas. (A) Sketch of the IST nanodisk array geometry, infrared illumination, and the optical crystallization of the antennas. (B) Simulated normalized absolute electric fields of the IST nanodisks in resonance in the cross-section plane at half-height of the IST core. The scale bar is 500 nm long. The antenna boundary is shown with a white circle. (C) Measured and (D) simulated transmittance spectra of the disk antenna array in its aIST (red) and cIST (blue, optically crystallized) states. The dashed black lines in the plots at the *x*-axis mark the position of the first order grating resonances through air and CaF_2_ (an average angle of incidence of 15° is assumed).

We fabricated a disk antenna array by focused ion beam milling. The experimental setup is sketched in Figure 3A. The IST disks are arranged in a square lattice with a total size of about 20 × 20 µm^2^, containing 9 × 9 antennas with a period of *p* = 2.1 µm. The disks have a height equal to the whole layer stack thickness (approx. 280 nm, see Figure S3, Supplementary Information) and a diameter of *D* = 1.7 µm. They are excited with light polarized in *y*-direction.

We simulated the absolute normalized electric fields in the cross-section at half-height of the IST core (see Figure 3B). Similar to the previously discussed IST rod antennas, aIST and cIST disk antennas fundamentally differ in their near-field distribution. For aIST, the field is concentrated at the center of the disk as well as outside along the polarization direction. For cIST, there is practically no electric near-field within the disk. Only the hotspots at the edge of the disk in polarization direction remain. The field enhancement is slightly reduced compared to the dielectric antenna which might be due to the increased optical losses in the crystalline IST phase.

Figure 3C displays the measured infrared transmittance spectra of the disk antenna arrays. In the amorphous phase (red) the transmittance has a narrow resonance minimum at a wavelength of *λ*_res_ = 3.62 µm and a width of FWHM = 0.24 µm. The *Q*-factor computes to *Q* = *λ*_res_/FWHM = 15.2. The small kink at about 4.3 µm is due to atmospheric CO_2_ absorption and another kink at a wavelength of $\lambda_{\mathrm{air}}=2.8$ µm stems from the first order grating resonance through air. The first order grating resonance through the CaF_2_ substrate is situated at a wavelength of $\lambda_{\mathrm{Ca}F_{2}}=3.6$ µm and overlaps with the resonance minimum of the sharp antenna resonance. The IST disks were subsequently optically crystallized (see **Materials and Methods**). The resulting infrared spectrum of the cIST antennas (blue) shows a significantly wider resonance with FWHM = 1.98 µm. As designed, the broad transmittance minimum is “pinned” at the grating resonance of $\lambda_{\mathrm{Ca}F_{2}}=3.6$ µm, resulting in a drastically reduced resonance quality with *Q* = 1.9. The small kink caused by CO_2_ absorption is also visible in the cIST spectrum.

The experimental spectra were again compared to simulated transmittance spectra (see Figure 3D) which were angle-averaged to approximate the light incidence through a Cassegrain objective in the experiment. The simulated spectra are in relatively good agreement with the measured spectra. Again, the grating resonances at 2.8 µm and 3.6 µm are overemphasized compared with the 9 × 9 disk array in the experiment. Therefore, the plasmonic resonance (blue) is located at slightly larger wavelength than the dielectric resonance (red). The minima at a wavelength of about 3 µm are much less pronounced in the measured spectra than in the simulations. In addition to the infinitely large array size in the simulations (see **Materials and Methods**), this could be caused by the range of incident angles of the Cassegrain objective, the thermal light source of the FTIR with weak coherence [56] or slight fabrication imperfections (see Figure 1C).

Interestingly, there is also a small feature at a wavelength of 4.3 µm in the simulated transmittance spectrum for aIST. This feature overlaps with the CO_2_ absorption dip in the experimental spectrum in Figure 3C. This is evidence that an assignment and interpretation of resonance modes in nanoantennas based on measured spectra can prove difficult and lead to overlooking of certain modes (high-*Q* modes especially). To gain more insight into the resonant modes in the amorphous phase of the nanoantennas, we calculated their quasinormal modes (QNMs, see Supplementary Note 4, Supplementary Information) [57], [58], [59]. The complex eigenfrequencies of QNMs represent both the resonance frequency (real part) and the resonance width (imaginary part) and are thus well-suited for describing the resonance tuning of the IST antennas. For the case of aIST disk resonator arrays in particular, the QNM analysis allowed us to identify the resonant modes of the structure at frequencies below the first diffraction order and immediately explain the field distribution in Figure 3B as arising from the fundamental dipole mode of the structure (see Figure S4, Supplementary Information). Moreover, our analysis revealed two other high-*Q* modes, one of them at higher and one at lower wavelength than the fundamental dipole resonance. These two modes can only be excited at non-normal incidence when the symmetry of the system is broken. The noted mode at about 4.3 µm corresponds to an out-of-plane magnetic dipole with a characteristic in-plane ring-shaped electric field distribution inside of the antenna. The other mode at shorter wavelengths than the dipole mode but still larger wavelength than the grating resonance is even harder to spot in the spectra. It is probably the origin of the small kink in the measured spectrum at about 3.1 µm and in the simulated spectrum at about 3.2 µm.

### 2.3 Resonance evolution in IST disk antennas with increasing crystallization depth

**Figure 4:**
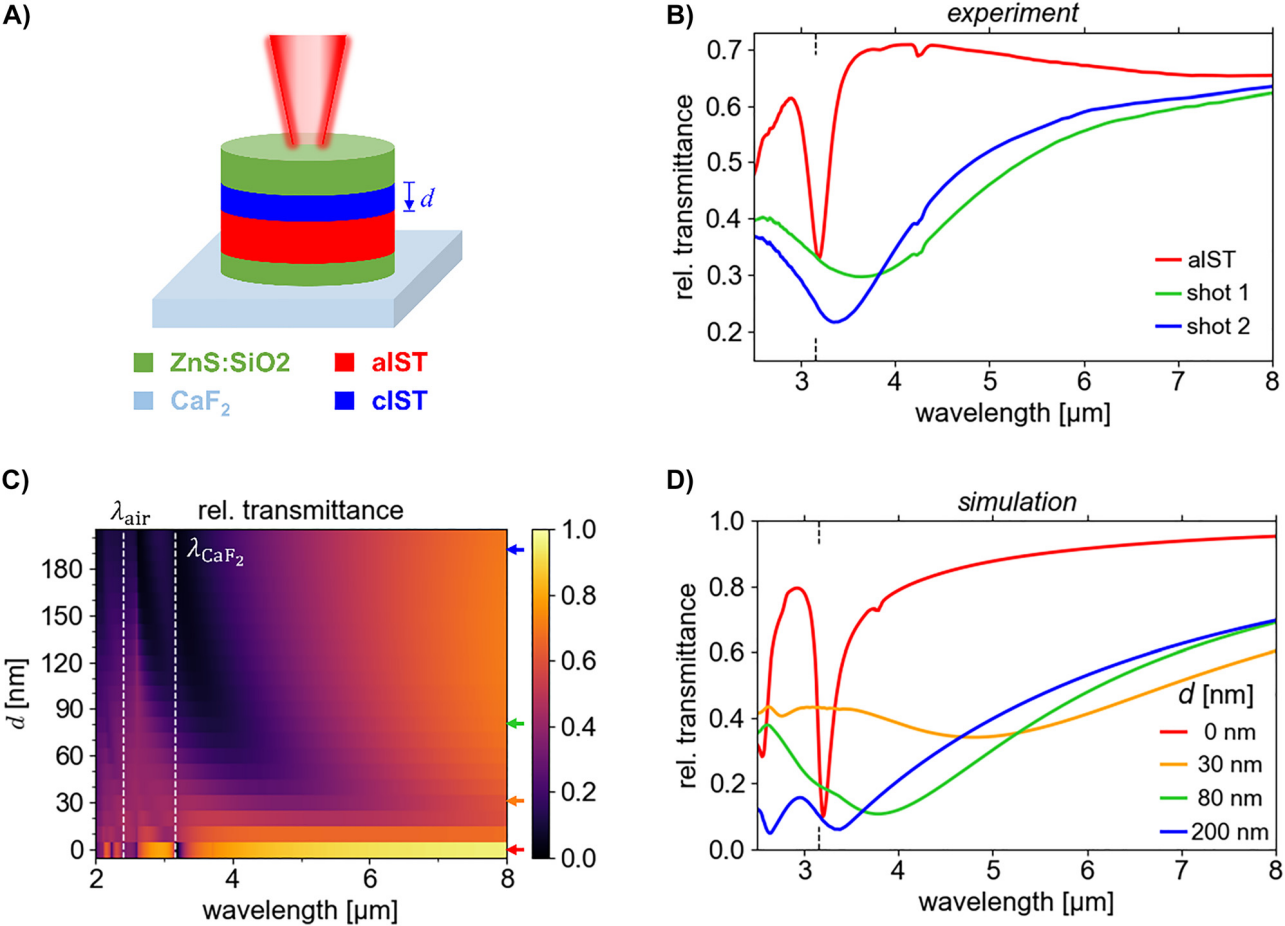
Crystallization depth tuning by optical switching in IST nanodisks. (A) Sketch of the layer stack of disk antennas (*D* = 1.5 µm, *p* = 1.9 µm): by optically switching the antenna from the top with a pulsed laser, the aIST is crystallized from top-to-bottom to a crystallization depth *d*. Successive laser shots with increased pulse power lead to an increase of the crystallization depth *d*. (B) Measured transmittance spectra of aIST disk arrays (red) and the same disks after the first (green) and second (blue) laser shot. (C) Simulated transmittance spectra of the disk antenna array for crystallization depths *d* from 0 nm (aIST) to 200 nm (cIST). The grating resonances in air and CaF_2_ are marked with dashed white lines. (D) Angle-averaged simulated transmittance spectra for different crystallization depths *d* (also indicated by the colored arrows in panel C). The grating resonance wavelength at 3.15 µm is marked in the experimental and simulated spectra with a dashed black line on the *x*-axis.

Up to now, only full crystallization was demonstrated. As shown in literature, however, it is possible to achieve intermediate steps between fully amorphous and fully crystalline states [35]. It was previously shown that optical switching crystallizes the PCM from the top capping towards the bottom substrate [42] because of the drastically different thermal properties of the air (bad thermal conductor) and the substrate (better thermal conductor). The resulting temperature gradient leads to the crystallization from the top of the PCM layer down to a certain crystallization depth. Therefore, we also investigated the optical switching of the IST disks in more detail and evaluated the crystallization depth *d* within the disks (see Figure 4A) by comparing experimental and simulated spectra.

For this, we fabricated a second IST disk array with antenna diameters of *D* = 1.5 µm and period of *p* = 1.9 µm. We realized an intermediate switching state in the IST disks by reducing the pulse power of the switching laser (see **Materials and Methods**). The measured infrared spectra are displayed in Figure 4B. In the initial state, the aIST disk antennas again display a sharp minimum in the transmittance spectrum (red) at a wavelength of approx. 3.18 µm. After the first crystallization shot (one shot consists of two equal pulses) at 3 mW laser pulse power, the intermediate state (green) is achieved with a redshifted, relatively broad resonance minimum at a wavelength of about 3.64 µm. Such a broad resonance indicates significant optical losses typical for plasmonic materials. This fits to the hypothesis that the top part of the aIST disk is crystallized in this state. After switching the disk antennas again with a second shot of larger laser pulse power of 8 mW (blue), the cIST antenna resonance blueshifts again to a resonance wavelength of 3.34 µm which is close to the initial resonance position for the aIST disks. Additionally, the resonance width decreases slightly. The feature at a wavelength of 4.3 µm is again due to atmospheric absorption by CO_2_. The feature at a wavelength of about 3.8 µm is due to the out-of-plane magnetic dipole (see above).

Next, numerical simulations of the aIST disks with increasing crystallization depth *d* were performed. The disk antennas were excited with s-polarized light and an angle of incidence of 15° (approximating the center of the angular intensity distribution of the Cassegrain objectives). In the colorplot of the simulated transmittance spectra (see Figure 4C), a sharp minimum at about 3.2 µm can be identified for completely amorphous IST (*d* = 0 nm). This is the high-*Q* resonance of the dielectric aIST antennas. For increasing crystallization depth *d*, this resonance blueshifts and its amplitude decreases until it is gone at about *d* = 40 nm. Such a behaviour is expected for dielectric disk antennas with decreasing antenna height (see Figure S5B, Supplementary Information). Simultaneously, a much wider resonance emerges at longer wavelengths of about 5 µm. This is the plasmonic resonance of the growing cIST disk at the top of the antenna. This resonance also blueshifts with increasing *d* until it overlaps with the first order grating resonances at 2.3 µm ( $\lambda_{\text{air}}$ ) and 3.15 µm ( $\lambda_{{\text{CaF}}_{2}}$ ). This is again expected for metallic disk antennas with increasing antenna height (see Figure S5C, Supplementary Information).

In Figure 3D, simulated transmittance spectra are displayed for several crystallization depths *d*. They are again averaged according to the angular intensity distribution of the Cassegrain objective. The respective crystallization depths are also marked in Figure 3C by colored arrows. The simulated spectra fit well to the measurements in Figure 3C. In comparison to the experimental spectra, the simulations suggest that the intermediate crystallization state caused by the first shot might have a crystallization depth of about *d* = 80 nm. Similarly, the measured resonance after switching with the second shot fits well to a crystallization depth of *d* = 200 nm.

These first proof-of-principle investigations thus strongly indicate that the optical crystallization of the IST antennas proceeds with an increasing crystallization depth *d*. Moreover, the measured transmittance spectra cannot be explained with an effective medium approach where the permittivity of amorphous and crystalline IST within the disks is averaged according to the crystallization ratio (see Supplementary Note 6). Further work on the confirmation of this hypothesis is needed, however: On the one hand, analysis of cross-section lamellas with transmission electron microscopy was previously used to measure the crystallization depth in an unpatterned PCM layer [42], but fabrication of such lamellas is very challenging for the nanostructured arrays. On the other hand, multiphysics simulations [42, 60, 61] could be used to model the crystallization and confirm the evolution of the crystallization depth.

## 3 Conclusion and discussion

In_3_SbTe_2_ (IST) is emerging as a promising new phase-change material (PCM) for infrared nanophotonics. Its special nature enables the switching of its infrared optical properties between a dielectric amorphous (aIST) and plasmonic crystalline (cIST) phase. We experimentally and numerically demonstrated the resonance tuning of nanostructured IST antennas. By switching the IST with laser pulses, we could change the type of the infrared resonances from sharp Mie resonances (aIST) to broad plasmonic resonances (cIST). With IST nanorod antennas, we could tune the antenna resonance wavelength and width at the same time. With IST nanodisk antennas, we showed the tuning of only the width of the resonance while leaving its position constant. Additionally, we realized an intermediate crystallization step by changing the crystallization depth within the disk antennas. Our work points towards new design concepts for active nanophotonic components, allowing the tuning of function and bandwidth at the same time. Possible applications in the infrared spectral range include active spectral filters, tunable absorbers and switchable flat optical devices like beam steerers and lenses.

IST is a representative of a new class of non-volatilely switchable, plasmonic PCMs [29]. Thus, the presented concepts should be applicable to other such plasmonic PCMs, like AgSnTe_2_. The performance may even be improved by, for example, using a plasmonic PCM with larger optical losses for the tuning of the resonance width.

Because thermal transport in the layer stack is not optimized, it is not possible to achieve the quenching rates required for reamorphization (>1 K/ns). As a solution, one could, for example, substitute the CaF_2_ substrate with another substrate of larger thermal conductivity. When using silicon instead of CaF_2_, the thermal conductivity of the substrate is increased by a factor of more than 10, which is sufficient to reach cooling rates which are high enough for reamorphization (see Supplementary Note 7). Alternatively, one could reduce the thickness of the IST layer by sandwiching it between two germanium layers (with a similar refractive index to aIST) to ease reamorphization [43, 51].

The presented quasinormal mode (QNM) analysis proved useful in determining the underlying resonant modes in the aIST disk antennas and recognizing and correctly interpreting small features, which could otherwise be easily overlooked in the measured and simulated spectra. A QNM analysis can in principle be performed also for the crystalline phase, although the relatively large decay rates make it difficult to calculate the complex resonance frequencies to sufficient accuracy. For the disk resonators in the present case, however, such an analysis is further complicated by the excitation of diffracted waves close to or beyond the first grating resonance. A thorough investigation of this region of the spectrum in terms of QNMs represents an exciting and challenging possibility for future research.

Another promising avenue of research may be applying the presented concepts to guided-mode resonators [62] or to antenna arrays which host optical bound states in the continuum (BICs) [63]. While the dielectric resonances considered here already have a high resonance quality, quality factors on the order of 10^3^ were achieved with such BICs [64]. Switching between the lossless dielectric and lossy plasmonic phases of IST could have an even bigger impact in such systems.

Going beyond simple resonance tuning, with IST it becomes possible to fundamentally change the type of the resonance and the corresponding near-field distributions. The corresponding change in electric field enhancement and hotspot location could be well-suited for switchable (bio)sensing applications based on surface-enhanced infrared absorption (SEIRA) [65]. The changes in the resonance width could be used to change the sensing from spectrally specific (narrowband SEIRA) to spectrally relatively unspecific (broadband SEIRA).

Finally, the on/off switching of the plasmonic resonances in the IST rod antennas investigated should enable straightforward application in switchable metasurfaces based on the geometric phase, as recently shown for conductive polymer antennas [20] with volatile electrical switching. Now, this concept can also be realized with all-optical, non-volatile switching. This is demonstrated numerically in Supplementary Note 8. The local optical switching of individual IST antennas should furthermore allow the integration of multiple functionalities on the same metasurface [25], facilitating optical programming of each functionality by turning the required antennas on or off.

## 4 Materials and Methods

### 4.1 Sample fabrication

Onto a CaF_2_ substrate by CrysTec GmbH, 10 nm (ZnS)_80_:(SiO_2_)_20_, 200 nm In_3_SbTe_2_ (IST) and 70 nm ZnS:SiO_2_ were sputtered. IST was deposited by direct magnetron sputtering, while ZnS:SiO_2_ was deposited with radio frequency magnetron sputtering. An LS 320 von Ardenne system (base pressure $1.6\cdot 10^{-6}$ mbar, 20 s.c.c.m. Ar flow, deposition rates of 1 Å/s for IST and 0.3 Å/s for ZnS:SiO_2_) was operated in constant power mode (20 W for IST and 59 W for ZnS:SiO_2_) using stochiometric targets of 99.99% purity. Next, a 60 nm-thick layer of the conductive polymer Electra 92 by Allresist GmbH was deposited by spin coating with the system SCE 15 by Schaefer Tec with 35 rps for 1 min at 15 s ramp time. The polymer layer was baked at 90 °C for 2 min afterwards. Subsequently, the nanoantennas were patterned with focused ion beam milling (FIB) with a Helios NanoLab DualBeam 650 by FEI company at a base pressure of $1\cdot 10^{-6}$ mbar, an ion beam current of 0.23 nA and an acceleration voltage of 30 kV. After FIB, the Electra92 polymer is removed again by submerging it in distilled water for 2 min. Any remaining resist is finally removed by plasma cleaning the sample at 20 W for 2 min at a pressure of 0.32 mbar.

The rods in Figure 2 have periods of *p*_*x*_ = 1.6 µm and *p*_*y*_ = 2 µm in *x*- and *y*-directions, respectively. The antennas are *w* = 0.4 µm wide and have a length of *L*_1_ = 0.9 µm and *L*_2_ = 1.6 µm, as indicated in the figure. The disk antenna array in Figure 3 has a period of *p* = 2.1 µm and a disk diameter of *D* = 1.7 µm, while in Figure 4 has a period of *p* = 1.9 µm and a disk diameter of *D* = 1.5 µm.

### 4.2 Measurement details

For scanning electron microscopy (SEM), a beam current of 100 pA and an acceleration voltage of 5 kV were used directly after antenna fabrication with the Helios NanoLab DualBeam 650 by FEI company. The Fourier transform infrared (FTIR) spectroscopy data was collected with a Hyperion 2000 microscope connected to a Vertex 70 spectrometer, both by Bruker. The spectra were measured through a 15× Cassegrain objective with linearly polarized light at a spectral resolution of 16 cm^−1^ (corresponding to approx. 10 nm in wavelength units), averaging over 1000 scans. The incident angles of the Cassegrain objective range from about 10° to 24°. This leads to a broadening of grating resonance and antenna resonance features in the measured transmittance spectra [54]. The antenna arrays were selected in the view field by a knife edge aperture. The spectra are normalized to the transmission through an area of the same size on a bare CaF_2_ substrate.

### 4.3 Laser switching

The IST antennas were switched optically with an in-house-built laser setup [42]. A pulsed laser beam (wavelength *λ* = 660 nm) is focused through a 10-fold objective with a numerical aperture of 0.25 on the sample surface. The sample is placed on a Thorlabs NanoMax-TS (Max311/M) stage, which is movable in *x*-, *y*- and *z*-direction and connected to a Thorlabs closed-loop piezo controller (BPC303). A custom program allows for the automated positioning of pulsed laser shots on the sample surface within 5 nm accuracy. Each antenna in an antenna array is switched individually by targeting them one after another with the switching laser. For crystallization, the IST has to be heated above its crystallization temperature of approximately 292 °C [66]. For reamorphization, it has to be heated above its melting temperature of approximately 626 °C [66] and then quenched rapidly (typically with cooling rates >10^9^ K/s).

The rod arrays in Figure 2 were crystallized with laser shots consisting of two pulses, separated by 0.5 s, with a pulse power of 3 mW and a pulse duration of 1.1 µs. The disk antennas in Figure 4 were only partially crystallized with a shot (2 pulses with 1.1 µs pulse duration) at pulse power of 3 mW. After an additional shot with increased pulse power of 8 mW, the IST antennas are (almost) completely crystallized. The disk antennas in Figure 3 are completely crystallized by using the two laser shots with 3 mW and 8 mW successively.

### 4.4 Simulations

Full-wave three-dimensional simulations were performed using a commercial solver, CST Studio Suite. Excitation by Floquet ports was used to model the experimental setup and to calculate the spectra. Unit cell boundaries were used in lateral directions. To approximate the spectra measured with the Cassegrain objectives, simulated spectra for angles between 10° and 24° were averaged according to the intensity distribution of the 15× objective [67]. The permittivity of IST is shown in Figure S1, the refractive index of CaF_2_ is approximated as 1.4 and that of ZnS:SiO_2_ as 2.1. The electric near fields in Figures 2 and 3 are simulated with normal incidence at the antenna resonances to better study the relevant near field patterns without distortion due to oblique incidence. Although the density of IST increases by about 8.6% upon crystallization [46], the influence of the corresponding maximum height change of about 17 nm on the transmittance spectra of the crystalline IST antennas is negligible (see Figure S5C, Supplementary Information).

For our QNM calculations (for more details see Supplementary Note 4, Supplementary Information), we used the open software packages gmsh [68] and getDP [69] to set up and solve Maxwell’s equations in a finite element framework. We largely follow the approach of Ref. [70], although, for simplicity, we model the open geometry by application of the Silver–Muller radiation condition on the top and bottom surfaces. To substantially simplify the calculations and the analysis, we consider here the case of perfectly normal incidence of the light. Similarly, we have partly neglected the 10 nm buffer layer, although the height of the buffer layer has been added to that of the IST resonator itself.

## Supplementary Material

Supplementary Material
