# Supplementary material for: Nanostructured In3SbTe2 antennas enable switching from sharp dielectric to broad plasmonic resonances
Source: Nanophotonics. 2022 Apr 25;11(17):3871–82. doi: 10.1515/nanoph-2022-0041 (PMC11501347; doi:10.1515/nanoph-2022-0041)
Supplement: Supplementary file 1 — Supplementary Material [file j_nanoph-2022-0041_suppl.docx]

Supporting Information for

**Nanostructured In_3_SbTe_2_ antennas enable switching from sharp dielectric to broad plasmonic resonances**

A. Heßler^*^, S. Wahl, P. T. Kristensen, M. Wuttig, K. Busch, T. Taubner^*^

*Corresponding authors. Email: hessler@physik.rwth-aachen.de; taubner@physik.rwth-aachen.de

**Table of Contents**

Supplementary Note 1: Infrared optical properties of In_3_SbTe_2_ (IST)

Supplementary Note 2: Simulated transmittance spectra of IST disk antennas under

normal incidence

Supplementary Note 3: SEM and AFM measurements of the IST disks

Supplementary Note 4: Quasi-normal mode simulations

Supplementary Note 5: Simulations of IST disks of different height

Supplementary Note 6: Effective medium approach

Supplementary Note 7: Alternative layer stack for reamorphization

Supplementary Note 8: Numerical demonstration of switchable beam steering

References

**Supplementary Note 1: Infrared optical properties of In_3_SbTe_2_ (IST)**

The following details about the optical properties of IST can be found in Ref. [1]:

The permittivity of IST can be described according to the model proposed by Shportko *et al.*.[2,3] The permittivity of the amorphous phase can be described by a Tauc-Lorentz oscillator model with the imaginary part:

| $\mathrm{Im}\left( \varepsilon_{TL}\left( \omega\right) \right)=\frac{A}{\omega}\frac{\omega_{0}\gamma\left( \omega-\omega_{g} \right)^{2}}{\left( \omega^{2}-\omega_{0}^{2} \right)^{2}+\gamma^{2}\omega^{2}}\Theta\left( \omega-\omega_{g} \right),$ | (1) |
| --- | --- |

where $\omega_{0}$ is the resonance frequency of the oscillator, $\gamma$ is the resonator damping, $A$ is the resonator strength, $\omega_{g}$ is the band gap frequency and $\Theta$ is the Heaviside function. The real part of the permittivity is then given by the Kramers-Kronig relations and an additional term $\varepsilon_{\infty}$ which accounts for the polarizability of the material in the higher frequency range.

For the crystalline phase permittivity, a Drude term must be added:

| $\varepsilon_{Drude}\left( \omega\right)=-\frac{\omega_{p}^{2}}{\omega\left( \omega+i\gamma_{D} \right)} ,$ | (2) |
| --- | --- |

where $\omega_{p}$ is the plasma frequency and $\gamma_{D}$ is the Drude damping.

The Tauc-Lorentz-Drude model parameters for amorphous and crystalline IST are summarized in **Table S1**. The permittivity is depicted in **Figure S1**.

**Table S1.** Tauc-Lorentz-Drude model parameters for IST.

|  | amorphous IST | crystalline IST |
| --- | --- | --- |
| $A$ [Hz] | 13.0⋅10^16^ | 4.0⋅10^16^ |
| $\omega_{0}$ [Hz] | 4.1⋅10^15^ | 4.1⋅10^15^ |
| $\omega_{g}$ [Hz] | 0.9⋅10^15^ | 0 |
| $\gamma$ [Hz] | 5.2⋅10^15^ | 4.1⋅10^15^ |
| $\omega_{p}$ [Hz] | - | 7.0⋅10^15^ |
| $\gamma_{D}$ [Hz] | - | 0.5⋅10^15^ |
| $\varepsilon_{\infty}$ | 2 | 1.4 |


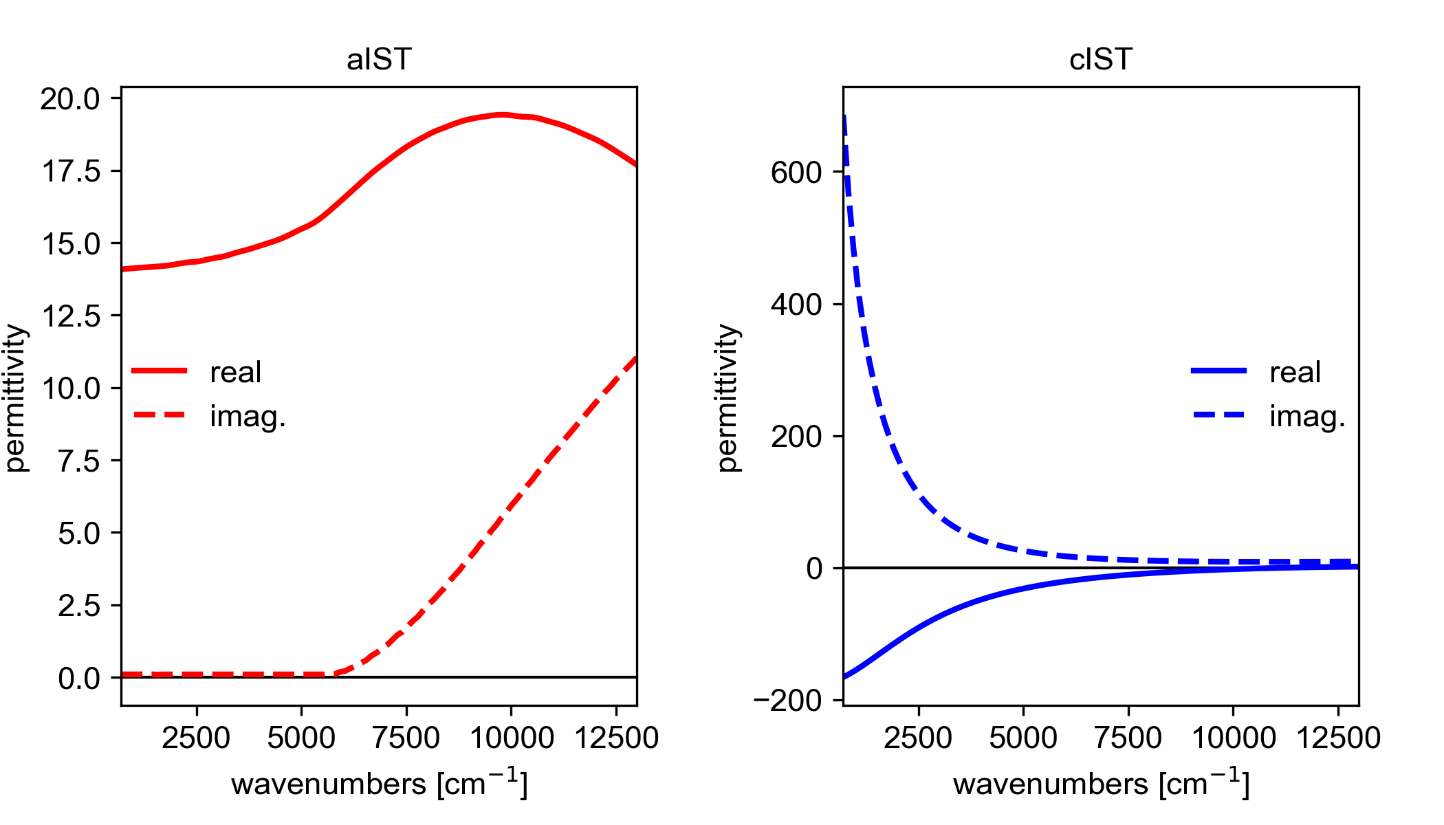


**Figure S1.** Permittivity of In_3_SbTe_2_ (IST). **Left:** Real (solid) and imaginary (dashed) part of the permittivity of amorphous IST (aIST). The real part is positive and the imaginary part zero. **Right:** Real (solid) and imaginary (dashed) part of the permittivity of crystalline IST (cIST). The real part is negative and follows a Drude-like behavior which is also visible in the imaginary part. The real part becomes negative at about 900 nm (11080 cm^-1^).[1]

**Supplementary Note 2: Simulated transmittance spectra of IST disk antennas under normal incidence**


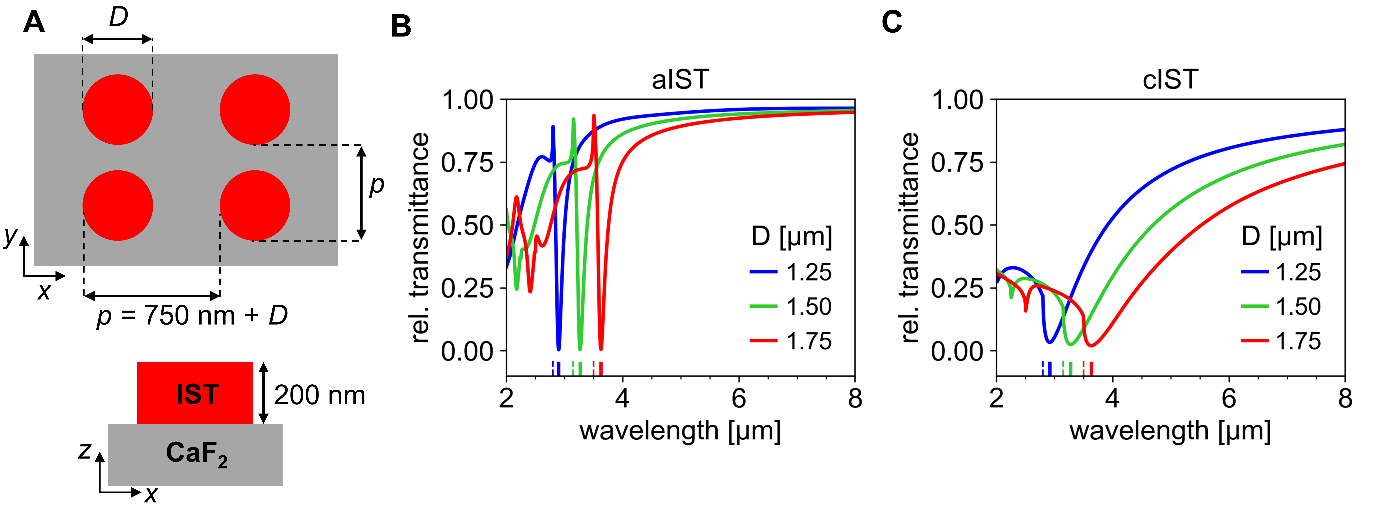


**Figure S2.** Simulated transmittance spectra of IST disk antennas (no capping) under normal light incidence. **A)** Sketch of the simulated geometry. The disks on the CaF_2_ substrate consist completely of IST, are 200 nm high and have a varying diameter *D*. The period *p* = 750 nm + *D* is adjusted such that the largest grating resonance wavelength is at only slightly smaller wavelength than the dielectric antenna resonances. **B)** The simulated transmittance spectra for aIST disks with different diameters *D* and periods *p*. There is a sharp minimum (marked with colored x-ticks) in each spectrum corresponding to the dielectric electric dipole resonance in the aIST disks. The position of the grating resonance ($\lambda_{CaF_{2}}=pn_{CaF_{2}}$) is demarked for each curve with a thin dashed line in the respective color. **C)** The simulated transmittance spectra for the same disks in the crystalline IST phase. The minimum corresponding to the plasmonic electric dipole resonance can clearly be seen. The broad minimum is “pinned” to the grating resonance. Therefore, the resonance minimum occurs at the same wavelength as in the amorphous phase.

**Supplementary Note 3: SEM and AFM measurements of the IST disks**


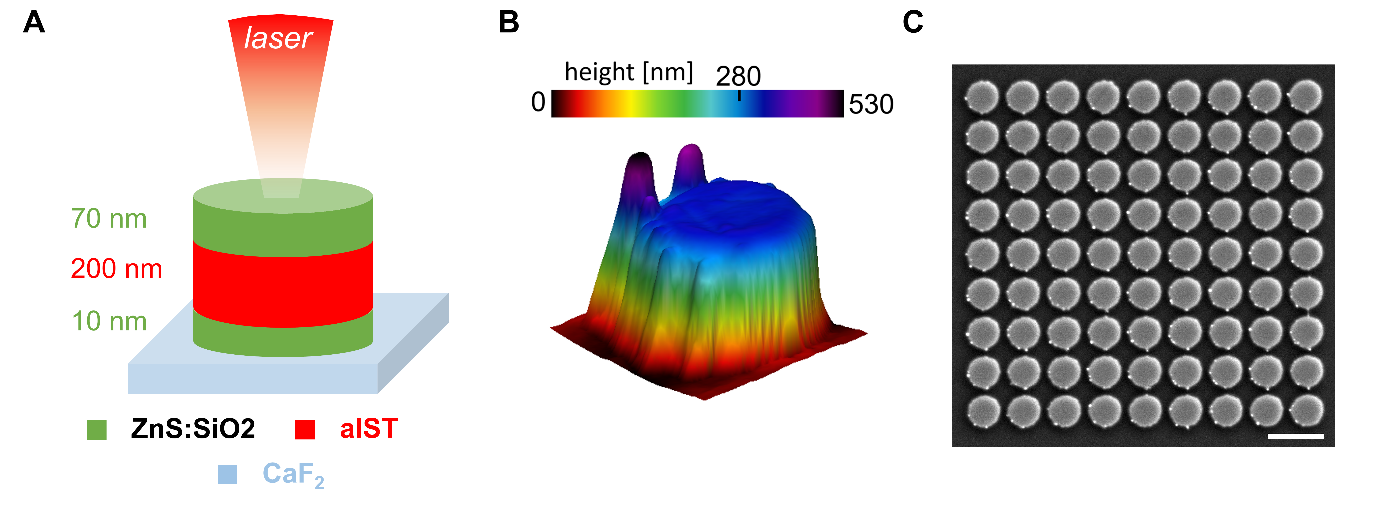


**Figure S3.** AFM and SEM images of the IST disk antenna array from **Figure 3** of the main text. **A)** Sketch of the layer stack of the IST disk antennas. **B)** AFM measurement of a disk antenna in a three-dimensional representation. **C)** SEM image of a disk antenna array. The scale bar equals 3 µm.

**Figure S3A** shows the sputtered layer stack of the IST disk antennas: 10 nm of ZnS:SiO_2_ below 200 nm of aIST capped by 70 nm of ZnS:SiO_2_ above, all on a CaF_2_ substrate. Not shown in the figure is that a conductive polymer needed to be applied on top of the layer stack to pattern it with focused ion beam milling. While most of this polymer can be removed in distilled water after processing, there can be some residue that does not dissolve because its molecular structure was damaged by the ion beam irradiation. We investigated the IST disks with atomic force microscopy (AFM). In **Figure S3B**, the measured height of the disk antennas is slightly larger than the expected 280 nm. On the one hand, this might be due to left-over residue of the conductive polymer on top of the antenna. On the other hand, this can be caused by milling away part of the CaF_2_ substrate in the fabrication process, effectively making the antennas appear higher. The “bunny ear” features at the edge of the disks which appear in the AFM image are probably caused by readsorption of milled material during focused ion beam milling. They are also visible in the SEM micrograph in **Figure S3C** as bright features. The SEM image shows the full array of disk antennas, i.e., the array consists of 9x9 = 81 antennas.

**Supplementary Note 4: Quasi-normal mode simulations**


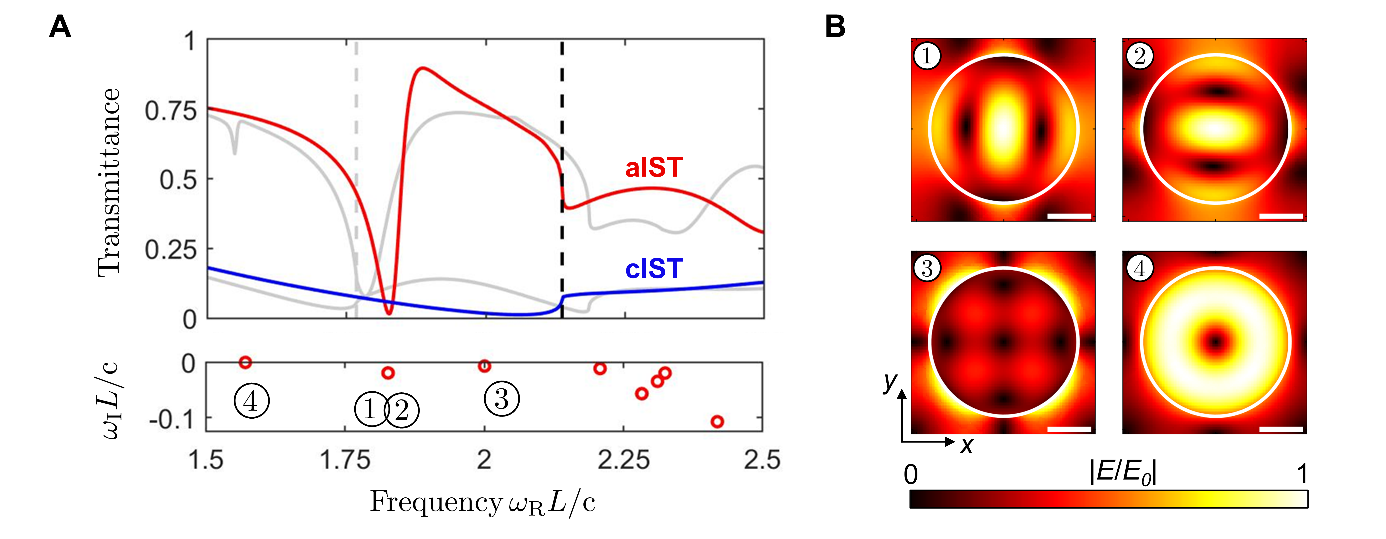


**Figure S4.** Quasinormal mode calculations for aIST disk antennas. **A)** **Top:** Normal incidence transmittance spectra for aIST (red) and cIST (blue) disk resonators. Vertical dashed line shows the first diffraction order at $\lambda_{\mathrm{Ca}F_{2}}=n_{\mathrm{Ca}F_{2}}p$, where *p* = 2100 nm is the period in both x- and y-direction. The non-normal incidence spectra corresponding to the simulations shown in **Figure 3** of the manuscript are also shown in gray. **Bottom:** Plot of the complex QNM frequencies, normalized to *c*/*L*, where *L* = 1 µm and *c* is the speed of light in free space. The imaginary part is shown on the y-axis and the real part on the x-axis. The frequencies of the four QNMs of interest are indicated by numbers, and the corresponding field profiles are shown in panel B. **B)** Field profiles of the four QNMs of interest in the amorphous phase with real part of the frequencies below the first diffraction order. As can be seen in the plot of the eigenfrequencies in panel A, modes 1 and 2 are two-fold degenerate.

To investigate the modal structure of the IST disk resonators more directly, we turned to the theory of quasinormal modes (QNMs)[4–6], which appear as solutions to the sourceless wave equation subject to a suitable radiation condition. The QNMs have complex resonance frequencies $\tilde{\omega}_{m}=\omega_{R}-i\omega_{I}$ with a negative imaginary part corresponding to a temporal decay of the field. The associated Q-value of a resonance can therefore be conveniently calculated as $Q_{m}=\frac{\omega_{R}}{2\omega_{I}}$. For our calculations, we used the open software packages gmsh[7] and getDP[8] to set up and solve Maxwell's equations in a finite element framework. We largely followed the approach of Ref. [9], although, for simplicity, we modeled the open geometry by application of the Silver-Muller radiation condition on the top and bottom surfaces. For most of the QNMs of interest, this resulted in small oscillations in the calculated frequencies as a function of calculation domain size, and the resulting numerical error on the calculated frequencies was found to be small compared to the main source of error stemming from the discretization. To substantially simplify the calculations and the analysis, we considered here the case of perfectly normal incidence of the light. Similarly, we partly neglected the 10 nm buffer layer, although the height of the buffer layer was added to that of the IST resonator itself.

The simulated transmittance spectra for normal incidence (colored) and oblique incidence (grey) for aIST and cIST are shown in the top of **Figure S4A**. The dashed lines mark the frequencies at which the grating resonances lie (black for normal incidence, grey for oblique incidence).

**Table S2:** Calculated complex eigenfrequencies and the corresponding wavelengths of the four QNMs indicated in **Figure S4**. The frequencies are normalized to *c*/*L*, where *L* = 1 µm nm and *c* is the speed of light in free space.

| **Mode number** | **1** | **2** | **3** | **4** |
| --- | --- | --- | --- | --- |
| **Re(**$\boldsymbol{\omega L/c}$**)** | 1.851 | 1.853 | 2.021 | 1.590 |
| **Im(**$\boldsymbol{\omega L/c}$**)** | -0.021 | -0.021 | -0.008 | -0.001 |
| **Re(**$\boldsymbol{\lambda}$**) [µm]** | 3.39 | 3.39 | 3.11 | 3.95 |
| **Im(**$\boldsymbol{\lambda}$**) [µm]** | 0.04 | 0.04 | 0.01 | 0.002 |

We focused on the QNMs for the amorphous phase, i.e., the aIST disks (see **Figure S4A**). We approximated the permittivities as real constants: $\epsilon_{\mathrm{aIST}}=14$ for aIST, and $\epsilon_{\mathrm{CaF}_{2}}=1.96$ for CaF_2_ and $\epsilon_{ZnS:\mathrm{SiO}_{2}}=5.0625$ for ZnS:SiO_2_. From analysis of the convergence, we found the complex resonance frequencies of the four fundamental modes at frequencies below the first grating resonance (see **Table S2**). In particular, we computed the frequency of the electric dipole mode in the amorphous phase as $\omega L/c=1.85 - 0.021i$, where *L* = 1 µm and *c* is the speed of light in free space. This corresponds to a (complex) wavelength of $\lambda=(3.39+0.04i)$ µm. Because of symmetry, this fundamental electric dipole mode of the aIST disk is two-fold degenerate (modes number 1 and 2). **Figure 3B** in the manuscript shows the field pattern of the y-polarized mode, which clearly resembles the field pattern in **Figure S4B**. This is no coincidence, since, in general, one can model the response of electromagnetic resonators by expansion into the QNMs.

For periodic gratings like the one we studied here[10–12], there may be additional contributions to the response at the various diffraction orders, which are not directly related to the QNMs. These effects, which are visible as sharp kinks in the transmission curves (see **Figure S4A**) can be handled by careful reformulation of the problem to include branch-cut contributions to the scattering matrix[12] or by extension of the solution space to include so-called PML modes[11]. This becomes especially relevant in the crystalline phase, where the optical response is governed largely by the excitation of diffracted waves above the first grating resonance (dashed black vertical line in **Figure S4A**). In the experiments, this was done so by design to “pin” the broad plasmonic transmittance minimum at the grating resonance. However, it prevents a simple interpretation in terms of a single or a few QNMs as discussed above and calls for a more elaborate treatment which goes beyond the scope of this work. For the present work, we therefore focused on the amorphous phase and the frequency range below the first diffraction order.

In addition to the fundamental dipole modes, there are two high-Q modes in the frequency range of interest (modes number 3 and 4). Because of symmetry, these modes do not affect the transmission at normal incidence, but they do show up as minima in the simulated spectra for oblique incidence, where the symmetry is broken (see the light gray lines in **Figure S4A**). Mode number 4 shows the characteristic electric ring field of an out-of-plane magnetic dipole mode.

Especially these high-Q modes can be easily overlooked when only interpreting the resonance modes based on the simulated spectra. With QNM analysis, these modes are immediately apparent, and their resonance frequency, width and field distribution can be precisely calculated.

**Supplementary Note 5: Simulations of IST disks of different height**


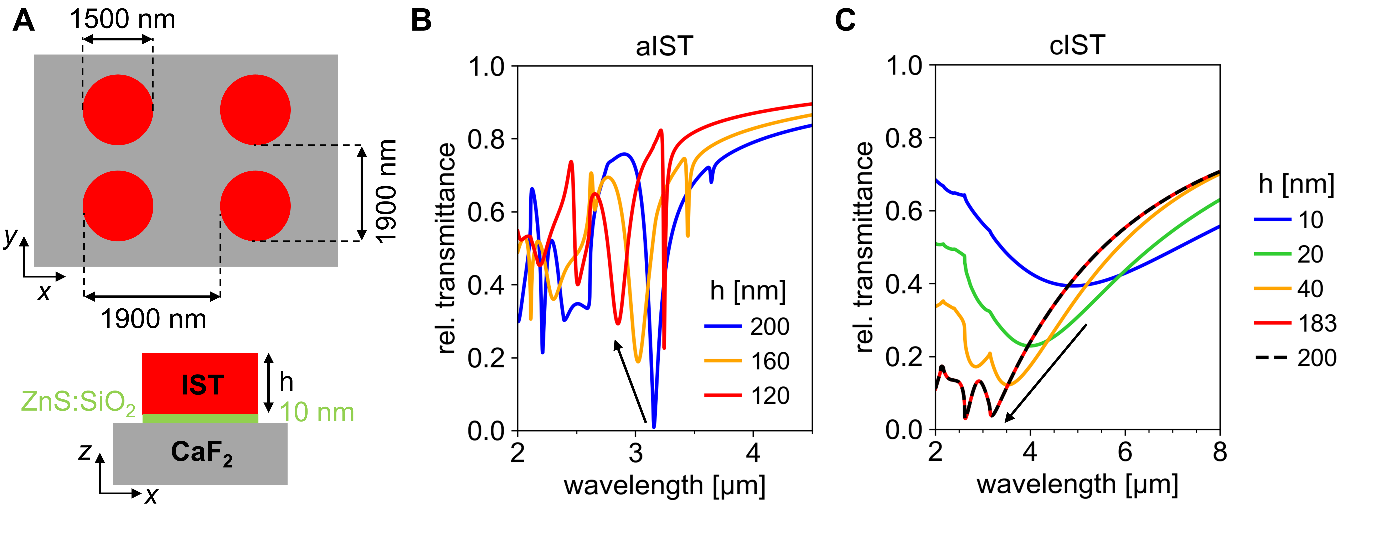


**Figure S5.** Simulations of IST disk resonances for different disk heights. **A)** Sketch of the simulated disk antenna geometry. The IST disks have a variable height *h*. **B)** Simulated transmittance spectra for aIST disks of different heights. With decreasing height *h*, the resonance minimum corresponding to the electric dipole blueshifts and decreases in amplitude. The sharp resonance at higher wavelengths originating from the out-of-plane magnetic dipole (see **Figure S4**) also blueshifts and increases in amplitude. **C)** Simulated transmittance spectra of cIST disks of different heights. With increasing height *h*, the broad resonance of the electric dipole blueshifts, increases in amplitude and becomes narrower. There is almost no difference between the transmittance spectra for heights of 183 nm and 200 nm. Thus, the increase of about 8.6% in the density of IST upon crystallization is negligible.

**Supplementary Note 6: Effective medium approach**

To show that the measured transmittance spectrum of the intermediate crystallization state in **Figure 4B** of the main text cannot be explained with an effective medium approach, we calculated the permittivity of IST according to the Bruggeman mixing formula:

| $f\frac{\varepsilon_{c}-\varepsilon}{\varepsilon_{c}+2\epsilon}+\left( 1-f \right)\frac{\varepsilon_{a}-\varepsilon}{\varepsilon_{a}+2\varepsilon}=0,$ | (3) |
| --- | --- |

where *f* is the crystallization ratio, *ε*_c_ is the permittivity of crystalline IST, *ε*_a_ is the permittivity of amorphous IST and *ε* is the effective, averaged permittivity of the partially crystalline IST.

The resulting permittivity is shown in **Figure S6A-B**. The real part is negative in the considered infrared spectral range for *f* = 0.6 and larger. The corresponding simulated transmittance spectra are depicted in Figure S6C. The array and antenna geometries are the same as in **Figure 4** of the main text (indicent angle of 15°). When comparing **Figure S6C** with **Figure 4B**, it is apparent that the measured transmittance spectra cannot be explained with an effective medium approach. In contrast, the modeling approach considering a crystallization depth *d* in Figure 4 can reproduce the experimental data relatively well.


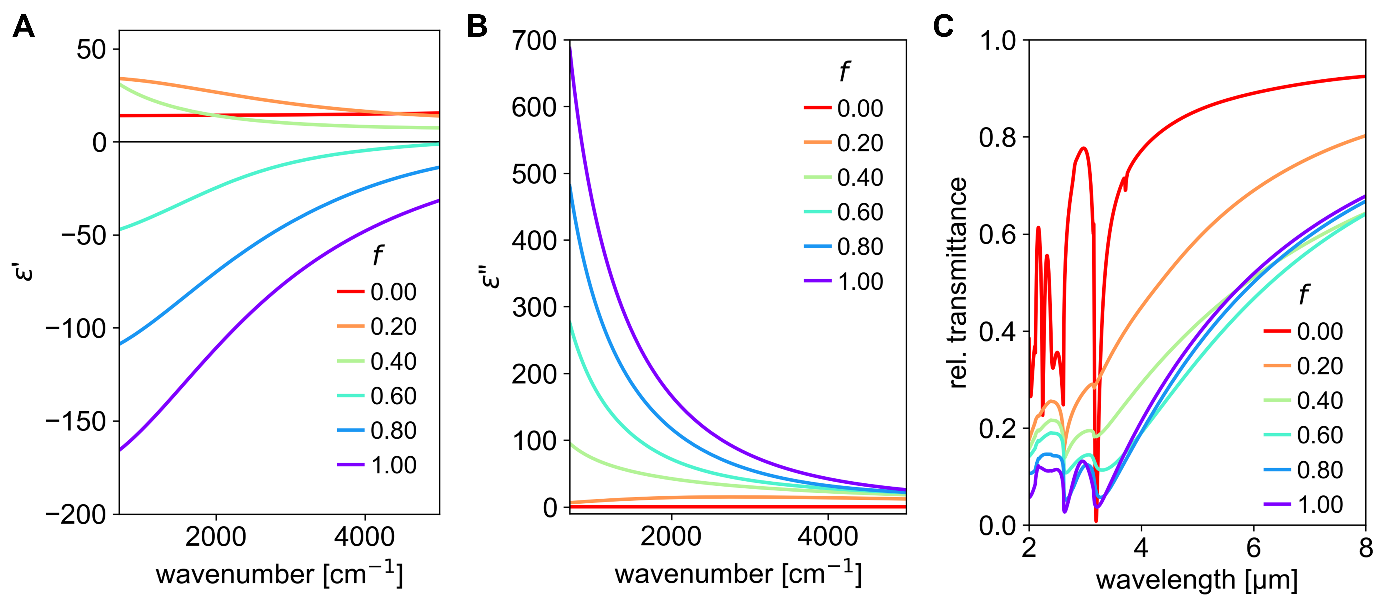


**Figure S6.** Simulations of intermediate crystallization steps in IST disks with a Bruggeman effective medium approach. **A)** Real part of the averaged permittivity of IST according to the Bruggeman mixing formula for different crystallization ratios *f* (see Equation (3)). **B)** Corresponding imaginary part of the permittivity for the same *f*. **C)** Simulated transmittance spectra for the IST disk antenna arrays from Figure 4 of the main text for an incident angle of 15°.

**Supplementary Note 7: Alternative layer stack for reamorphization**

The thermal conductivity in the layer stack can be increased by employing a substrate with larger thermal conductivity than CaF_2_. For example, silicon has a thermal conductivity which is more than ten times larger than that of CaF_2_. By substituting the CaF_2_ substrate with a silicon substrate (see **Figure S7A**), cooling rates sufficient for reamorphization (> 1 K/ns) can be reached. This was verified in an optical switching experiment on an otherwise unpatterned layer stack (see **Figure S7B**). First, identical crystalline spots were each created with 10 pulses of 11.8 mW power and 450 ns duration in the otherwise amorphous IST matrix. Next, these crystalline spots were reamorphized with single pulses of varying power and duration. The pulse power was varied from 300 mW to almost 340 mW and the pulse duration was changed from 14 ns to 20 ns. In **Figure S7B**, it can clearly be observed that the size of the dark area of the reamorphized region within the bright crystalline spots became larger with increasing pulse power and duration. The diameter of the largest reamorphized spot is about 1.5 µm. This demonstrates that reamorphization is possible within this modified layer stack.


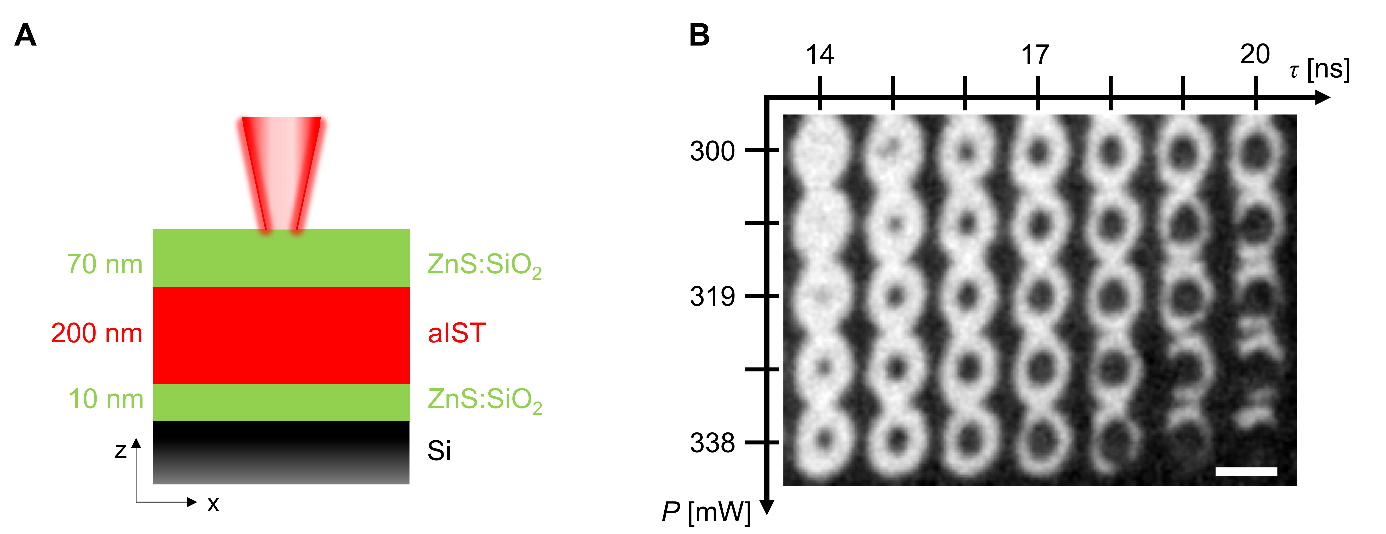


**Figure S7.** Alternative layer stack design for reamorphization. **A)** Sketch of the alternative layer stack design with a silicon substrate (higher thermal conductivity) instead of a CaF_2_ substrate. **B)** Light micrograph of a power-time-effect diagram for reamorphizing a crystalline spot in the layer stack. The scale bar is 2 µm long.

**Supplementary Note 8:** **Numerical demonstration of switchable beam steering**


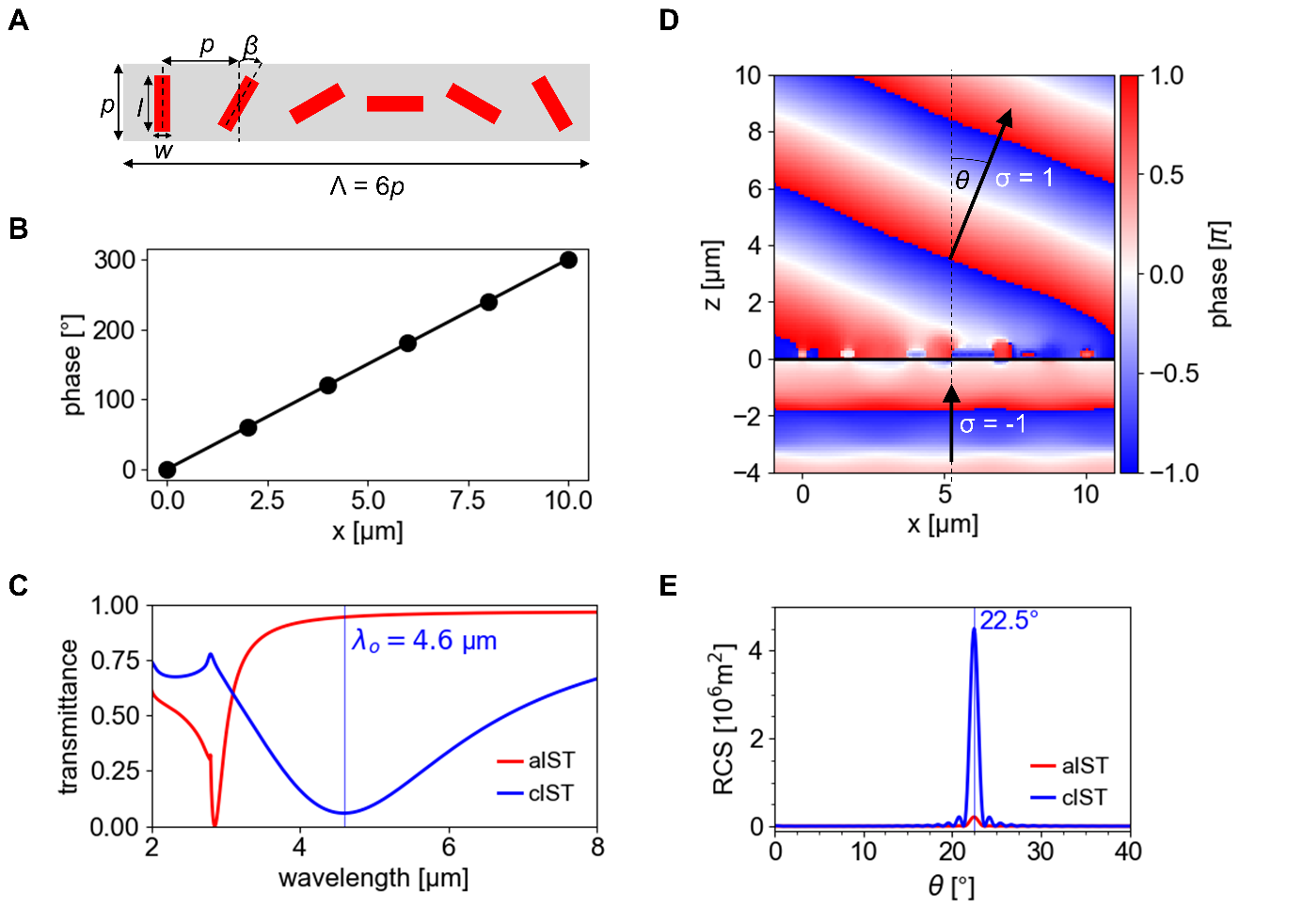


**Figure S8.** Switchable beam steering with IST rod antennas. **A)** Sketch of the super cell geometry consisting of 6 rod antennas of length l = 1600 nm, width w = 400 nm and period p = 2000 nm. The IST rod antennas are rotated by the angle *β* = 30° to impose a linear geometric phase gradient. **B)** Plot of the geometric phase (2*β*) against the x-position of the antennas in the super cell. **C)** Transmission spectra (normal incidence) of the aIST and cIST rod antennas without a geometric phase. The resonance wavelength of λ_o_ = 4.6 µm of the cIST rods is chosen as the operation wavelength of the beam steerer. **D)** Electric-field phase distribution of the beam steerer for crystalline IST when excited with left-handed circularly polarized light (σ = -1) from the bottom under normal incidence. We plot the incident phase and anomalous refraction phase of the opposite handedness circularly polarized light in the two half spaces below and above the metasurface at z = 0 (see the black horizontal line). The right-handed circularly polarized light (σ = 1) is refracted anomalously under the angle θ = 22.5°. **E)** Far-field radar cross section for right-handed circularly polarized light against the polar angle *θ*. Fitting to the near-field plot in panel D), most of the light is transmitted under the angle *θ* = 22.5° to the surface normal in the crystalline IST phase. In contrast, approx. 20 times less light is transmitted under this angle in the amorphous IST phase.

For numerical demonstration of a switchable beam steering functionality, rotated IST rod antennas are arranged on a metasurface to create a linear gradient of geometric phase (see **Figure S8A**). When excited from the bottom (substrate side) with left-handed circularly polarized light, the transmitted right-handed circularly polarized light is refracted anomalously because the metasurface imparts an additional geometric phase 2*β* at each meta-atom, where *β* is the rotation angle of the rods. The resulting linear phase gradient for *β* = 30° is plotted in **Figure S8B**. In **Figure S8C**, transmittance spectra of rod antenna arrays with the same period and antenna length and width but with *β* = 0 for all rods are shown (like in **Figure 2** of the main text). The incident light is linearly polarized parallel to the rods. In this configuration, the resonances of the rod antennas are apparent in the transmittance spectra. The resonance at a wavelength of 4.6 µm in the crystalline IST phase vanishes in the amorphous phase. This provides the ability to switch this antenna resonance “on” (cIST) and “off” (aIST). In **Figure S8D**, the phase distribution of the circularly polarized electric near-field demonstrates the anomalous refraction under an angle *θ* of the circularly polarized plane wave which incides from the bottom. The resulting beam deflection in the far-field can be observed in **Figure S8E**. The radar cross section (RCS) of the right-handed circularly polarized light has a maximum at *θ* = 22.5° for both IST phases. This fits well to the theoretical value[13] $\theta_{theo}=\arcsin(\lambda_{o}/\Lambda)=\arcsin(4.6/12)\approx22.5^{\circ}$. Because the IST antennas are only resonant in the crystalline phase, the RCS maximum of the cIST antennas is about 20 times larger than that of the aIST antennas. Thus, the efficiency of the metasurface for beam steering can be changed dramatically by switching the IST between amorphous (“off” state) and crystalline (“on” state) phases. This principle of on/off switching of the geometric phase can be used for arbitrary phase distributions and extended to functionalities like lensing or holography. A similar concept was realized recently through volatile, electrical switching of PEDOT:PSS rod antennas[14]. It can now also be realized by non-volatile, all-optical switching of IST antennas.

**References**

[1] A. Heßler, S. Wahl, T. Leuteritz, A. Antonopoulos, C. Stergianou, C.-F. Schön, L. Naumann, N. Eicker, M. Lewin, T. W. W. Maß, M. Wuttig, S. Linden & T. Taubner. In_3_SbTe_2_ as a programmable nanophotonics material platform for the infrared. *Nature Communications* **12**, 924 (2021).

[2] Rausch, Pascal & Wuttig, Matthias. Investigations of binary and ternary phase change alloys for future memory applications. (RWTH Aachen University, 2012).

[3] K. Shportko, S. Kremers, M. Woda, D. Lencer, J. Robertson & M. Wuttig. Resonant bonding in crystalline phase-change materials. *Nature Materials* **7**, 653–658 (2008).

[4] E. S. C. Ching, P. T. Leung, A. Maassen van den Brink, W. M. Suen, S. S. Tong & K. Young. Quasinormal-mode expansion for waves in open systems. *Reviews of Modern Physics* **70**, 1545–1554 (1998).

[5] P. Lalanne, W. Yan, K. Vynck, C. Sauvan & J.-P. Hugonin. Light Interaction with Photonic and Plasmonic Resonances. *Laser & Photonics Reviews* **12**, 1700113 (2018).

[6] P. T. Kristensen, K. Herrmann, F. Intravaia & K. Busch. Modeling electromagnetic resonators using quasinormal modes. *Advances in Optics and Photonics* **12**, 612–708 (2020).

[7] C. Geuzaine & J.-F. Remacle. Gmsh: A 3-D finite element mesh generator with built-in pre- and post-processing facilities. *International Journal for Numerical Methods in Engineering* **79**, 1309–1331 (2009).

[8] P. Dular & C. Geuzaine. GetDP: a General Environment for the Treatment of Discrete Problems. https://getdp.info/.

[9] G. Demésy, A. Nicolet, B. Gralak, C. Geuzaine, C. Campos & J. E. Roman. Non-linear eigenvalue problems with GetDP and SLEPc: Eigenmode computations of frequency-dispersive photonic open structures. *Computer Physics Communications* **257**, 107509 (2020).

[10] T. Weiss, M. Mesch, M. Schäferling, H. Giessen, W. Langbein & E. A. Muljarov. From Dark to Bright: First-Order Perturbation Theory with Analytical Mode Normalization for Plasmonic Nanoantenna Arrays Applied to Refractive Index Sensing. *Physical Review Letters* **116**, 237401 (2016).

[11] A. Gras, W. Yan & P. Lalanne. Quasinormal-mode analysis of grating spectra at fixed incidence angles. *Optics Letters* **44**, 3494–3497 (2019).

[12] A. B. Akimov, N. A. Gippius & S. G. Tikhodeev. Optical fano resonances in photonic crystal slabs near diffraction threshold anomalies. *JETP Letters* **93**, 427 (2011).

[13] N. Yu, P. Genevet, M. A. Kats, F. Aieta, J.-P. Tetienne, F. Capasso & Z. Gaburro. Light Propagation with Phase Discontinuities: Generalized Laws of Reflection and Refraction. *Science* **334**, 333–337 (2011).

[14] J. Karst, M. Floess, M. Ubl, C. Dingler, C. Malacrida, T. Steinle, S. Ludwigs, M. Hentschel & H. Giessen. Electrically switchable metallic polymer nanoantennas. *Science* **374**, 612–616 (2021).
